# Superimposed Neuroinvasive Coccidioidomycosis and West Nile Virus Infection

**DOI:** 10.7759/cureus.29783

**Published:** 2022-09-30

**Authors:** Leah M Grant, Sabirah N Kasule, Madeline L Singer, Lisa J Speiser, Holenarasipur R Vikram

**Affiliations:** 1 Infectious Disease, Mayo Clinic, Phoenix, USA; 2 Infectious Disease, BronxCare Health System, New York City, USA; 3 Neurology, Barrow Neurological Institute, Phoenix, USA

**Keywords:** arizona, west nile virus, neuroinvasive, meningitis, coccidiomycosis, flaviviruses

## Abstract

A 58-year-old man with recently diagnosed coccidioidal meningitis presented to the ED with a five-day history of headache, photopsia, blurred vision, and worsening encephalopathy. His coccidioidal meningitis had responded well to fluconazole therapy, but three weeks later, he developed acute symptomatic worsening. Unfortunately, his clinical worsening coincided with Arizona's worst seasonal West Nile Virus (WNV) outbreak. He was ultimately found to have WNV neuroinvasive disease. Concurrent coccidioidal and WNV neuroinvasive diseases have not been described in the literature. Fortunately, he improved quickly to his normal baseline without neurologic deficits with supportive therapy for his WNV neuroinvasive disease and remains on lifelong antifungal therapy for coccidioidal meningitis.

## Introduction

Arizona is home to the endemic dimorphic fungus *Coccidioides* immitis/posadasii, the etiologic agent of Valley fever most commonly manifests as pneumonia. Patients who reside in and visit Arizona are at risk for contracting Valley fever, which disseminates to the central nervous system (CNS) in less than 1% of infected individuals, causing meningitis [1]. West Nile Virus (WNV) has been present in Arizona since 2003, and yearly case counts range from 20 to 150, averaging around 100 cases yearly. In 2021, there were 1,567 cases of probable and confirmed WNV reported to the Arizona Department of Health and Services, compared to 11 cases in 2020 [2]. The significantly increased number of cases in 2021 has been attributed to a heavier monsoon season compared to prior years [3]. Therefore, clinicians should consider epidemiologic risk factors when evaluating patients with CNS infection.

## Case presentation

A 58-year-old man with no significant past medical history presented to an outside ED in Arizona during the month of August 2021 with a five-day history of headache, photopsia, blurred vision, and two days of nausea and vomiting. One month before this presentation, he developed a persistent non-productive cough. CT of the head was negative for evidence of acute stroke, intracranial arterial stenosis, or aneurysm. The patient improved with analgesic medications and was discharged home. Two weeks later (September 2021), the patient presented with acute onset diplopia to another facility. Repeat CT head was negative for acute intracranial abnormalities. Lumbar puncture was diagnostic of *Coccidioides* meningitis with a total nucleated cell count of 88 cells/μl (88% lymphocytes), protein of 160 mg/dL, positive *Coccidioides* IgM and IgG by ELISA, and complement fixation of 1:2. He was started on fluconazole 800 mg daily (standard initial therapy for coccidioidal meningitis) with slow improvement in his symptoms.

The patient's headache returned three weeks after starting fluconazole (early October 2021). He became progressively encephalopathic and was found by his wife at home, hallucinating and exhibiting strange behaviors. He was febrile to 38.3 ℃ and was brought to our institution for evaluation. On arrival at our ED, he appeared agitated, disoriented, and actively hallucinating. Physical examination was notable for diffuse hyperreflexia and myoclonus of the lower extremities. The presumptive diagnosis on admission was progressive coccidioidal meningitis either due to medication noncompliance, poor serum fluconazole levels, inadequate transfer across his blood-brain barrier, or development of a complication from coccidioidal meningitis (such as hydrocephalus, cranial vasculitis, or coccidioidoma).

CT scan of the head was again negative for acute intracranial abnormalities. Complete blood count was notable for a WBC count of 13.1 x 109/L. The complete metabolic panel was within normal limits. Due to his altered mental status, the patient underwent a repeat lumbar puncture which revealed a cerebrospinal fluid (CSF) nucleated cell count of 435 (87% lymphocytes, 9% monocytes, 3% eosinophils, and 1% neutrophils) with a total protein of 123 mg/dL and glucose of 34 mg/dL. A BioFire FilmArray Meningitis/Encephalitis panel was negative. The patient was admitted to the medical floor and started on vancomycin, ceftriaxone, ampicillin, and acyclovir. Fluconazole was continued. The infectious diseases team was consulted for coccidioidal meningitis.

**Figure 1:**
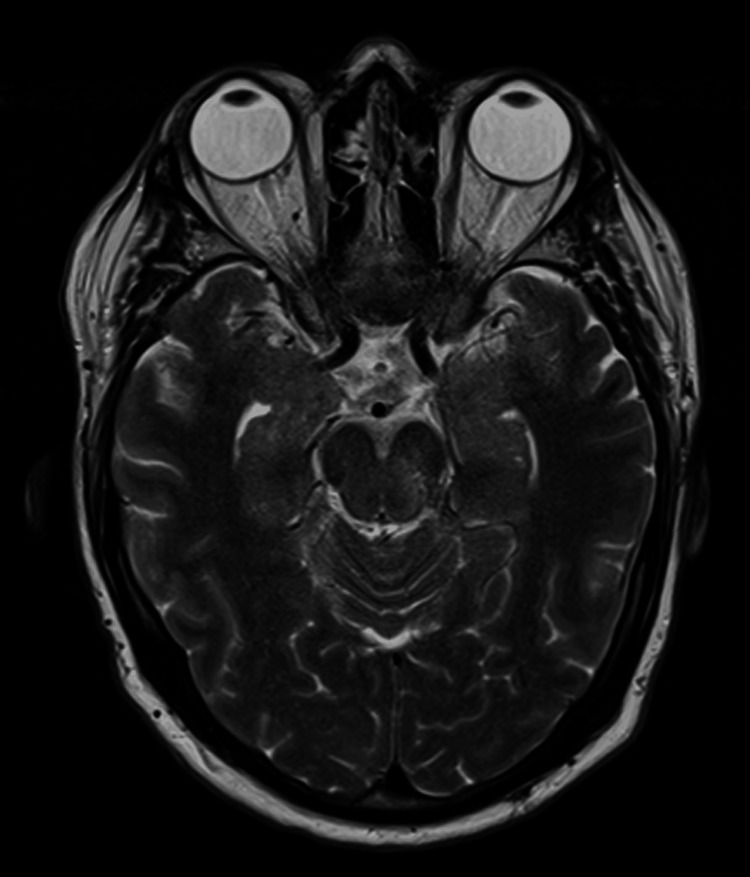
MRI brain showing areas of abnormal enhancement within the basilar cisterns (T2 FLAIR sequence, transverse plane). FLAIR: Fluid attenuated inversion recovery.

An MRI of the brain and spine was recommended to evaluate for structural complications of Coccidioides meningitis, including hydrocephalus and/or spinal arachnoiditis. Imaging findings were negative aside from areas of abnormal basilar cistern enhancement with mild adjacent intraparenchymal edema compatible with the patient's history of fungal meningitis (Figure 1). In addition, testing for WNV infection was recommended as the patient presented amid a WNV epidemic in Maricopa County, Arizona. Upon questioning, the patient's wife disclosed that the patient had sustained multiple recent mosquito bites.

The patient's CSF bacterial and fungal cultures were negative, as were blood cultures collected on admission. However, his WNV RNA polymerase chain reaction (PCR) from plasma and CSF and WNV IgM from serum and CSF returned positive, confirming a diagnosis of WNV neuroinvasive disease (Table 1).

**Table 1:** Patient's CSF results. CSF: Cerebrospinal fluid.

Patient’s CSF Results		
	September 2021	October 2021
Cell count	88	435
Differential	88% lymphocytes	87% lymphocytes
Protein	160	123
Glucose	N/A	34
Coccidioides serology by complement fixation	1:2	1:2
Bacterial and fungal cultures	Negative	Negative
BioFire FilmArray Meningitis/Encephalitis panel	N/A	Negative
WNV IgM	N/A	Positive

The patient returned to his baseline mental status with supportive care for West Nile infection and was discharged on hospital day four. He was continued on fluconazole 800 mg daily for known coccidioidal meningitis with planned infectious diseases outpatient follow-up. Further work-up was negative for evidence of underlying immunosuppressive disorders, including HIV and diabetes.

## Discussion

Coccidioidomycosis, the disease caused by *Coccidioides* immitis/posadasii, is an endemic fungal infection in the southwestern United States. The most common clinical manifestations are pulmonary and include cough, chest pain, and fever. In less than 1% of infected patients, the infection may disseminate to the skin, skeleton, and/or CNS [1]. Coccidioidal meningitis is a rare and serious complication of coccidioidomycosis, and if untreated, it is almost always fatal within two years of diagnosis. Meningitis usually develops within the first two years after infection, on average between 4 and 5 weeks from initial infection [4]. Our patient presented with a typical course of illness beginning with several weeks of non-productive cough with likely pneumonia, followed by symptoms of meningitis within weeks of his pulmonary *Coccidioides* infection. He was started on appropriate antifungal therapy and began to experience slow improvement in his symptoms. 

Since the arrival of WNV to Arizona in 2003, there have been yearly seasonal epidemics of the disease in Maricopa county (which includes Phoenix and Scottsdale). The yearly case count typically ranges from 50-115 per the Arizona Department of Public Health website, though in 2021, the statewide case count was over 1,000. Most of these cases occurred between the end of August 2021 and mid-October [2]. The cause of these record high case numbers has been attributed to increased rainfall during the monsoon season, leading to increased numbers of WNV-containing Culex mosquitoes [3]. While most WNV infections are asymptomatic, 20% of infected individuals will develop a fever. Other symptoms include chills, malaise, headache, arthralgias, nausea, vomiting, diarrhea, and a maculopapular rash. Neurologic involvement occurs in less than 1% of cases and can lead to encephalitis, meningitis, and/or neurologic deficits, including acute flaccid paralysis [5]. Out of Arizona's 1,567 reported cases of WNV infection in 2021, 924 patients presented with encephalitis, meningitis, or another manifestation of neuroinvasive disease. Sixty-eight patients in Arizona developed acute flaccid paralysis as part of WNV infection [2]. 

Our patient presented with fever, confusion, and headache in the setting of recently diagnosed coccidioidal meningitis. This was initially worrisome for worsened fungal meningitis or the development of a complication of the disease, such as hydrocephalus and/or spinal arachnoiditis. However, as his symptoms had initially responded well to antifungal therapy and the patient presented during an outbreak of WNV infection in Maricopa County, we believed the latter diagnosis was more likely. His diagnosis of WNV neuroinvasive disease was confirmed with serologic and CSF testing, and he improved with supportive care.

This case also highlights the importance of recognizing and avoiding cognitive biases such as anchoring and premature closure. Anchoring is the tendency to focus on a singular aspect of a case, which creates the potential to negate other disconfirming aspects of the case. Premature closure is the closure of the diagnostic process before all relevant information is obtained and, therefore, the failure to consider other diagnoses [6]. In our patient's case, his presenting symptoms led the team to anchor on the presumptive diagnosis of worsened coccidioidal meningitis, and premature closure prevented the team from initially considering alternative diagnoses such as WNV neuroinvasive infection.

## Conclusions

In conclusion, we describe a case presentation of coccidioidomycosis complicated by dissemination to the CNS, followed by superimposed viral meningoencephalitis. Our patient had initially improved while on antifungal therapy adequate for treatment of coccidioidal meningitis, and one month later presented with worsening symptoms. The worsening of symptoms while on therapy for coccidioidal meningitis raises several questions about the reason for treatment failure (structural complications, medication intolerance or noncompliance, etc.). As there was no evidence of worsened coccidioidal meningitis, a search for an alternative diagnosis was undertaken, and WNV neuroinvasive disease was diagnosed. This case report highlights the importance of considering epidemiologic risk factors and alternative diagnoses when evaluating patients with CNS infection.

## References

[1] Blair Blair, J J, Ampel Ampel, N N (2022). Coccidioidal meningitis. UpToDate.

[2] (2021). Arizona Department of Health Services. Arizona 2021 West Nile Virus Statistics. https://www.azdhs.gov/preparedness/epidemiology-disease-control/mosquito-borne/west-nile-virus/index.php.

[3] Rigler J (2022). Protect Yourself: Arizona is Experiencing a Record High Season for West Nile Virus. https://directorsblog.health.azdhs.gov/protect-yourself-arizona-is-experiencing-a-record-high-season-for-west-nile-virus/#:~:text=View%20Larger%20Image-,Protect%20Yourself%3A%20Arizona%20is%20Experiencing%20a%20Record%20High%20Season%20for,123%20cases%20and%20four%20deaths..

[4] Galgiani JN (2020). Coccidioidomycosis (Coccidioides Species). Mandell, Douglas, and Bennett's Principles and Practice of Infectious Diseases.

[5] Thomas SJ, Endy TP, Rothman AL (2020). Flaviviruses (Dengue, Yellow Fever, Japanese Encephalitis, West Nile Encephalitis, Usutu Encephalitis, St. Louis Encephalitis, Tick-Borne Encephalitis, Kyasanur Forest Disease, Alkhurma Hemorrhagic Fever, Zika). Mandell, Douglas, and Bennett's Principles and Practice of Infectious Diseases.

[6] Restrepo D, Armstrong KA, Metlay JP (2020). Annals clinical decision making: avoiding cognitive errors in clinical decision making. Ann Intern Med.

